# Detection of AmpC β-lactamase producing bacteria isolated in neonatal sepsis

**DOI:** 10.12669/pjms.326.10861

**Published:** 2016

**Authors:** Sonia Salamat, Hasan Ejaz, Aizza Zafar, Humera Javed

**Affiliations:** 1 Sonia Salamat, M.Phil. Department of Microbiology, Government College University, Lahore, Pakistan; 2 Hasan Ejaz, PhD, Department of Microbiology, The Children’s Hospital and The Institute of Child Health, Lahore, Pakistan; 3 Aizza Zafar, M.Phil, Department of Microbiology, The Children’s Hospital and The Institute of Child Health, Lahore, Pakistan; 4 Humera Javed, M.Phil, Department of Microbiology, The Children’s Hospital and The Institute of Child Health, Lahore, Pakistan

**Keywords:** AmpC β-lactamase detection, Antimicrobial resistance, Multidrug resistant bacteria, Neonatal sepsis

## Abstract

**Objective:**

The objective of this study was to determine the occurrence and antimicrobial profile of AmpC β-lactamase producing bacteria.

**Methods:**

The study was conducted at The Children’s Hospital and The Institute of Child Health Lahore, Pakistan, during September 2011 to June 2012. A total number of 1,914 blood samples of suspected neonatal septicemia were processed. Isolates were identified using Gram’s staining, API 20E and API 20NE tests. Gram negative isolates were screened for AmpC β-lactamase production against ceftazidime, cefotaxime and cefoxitin resistance and confirmed by inhibitor based method.

**Results:**

Total number of 54 (8.49%) Gram positive and 582 (91.5%) Gram negative bacteria were identified. Among Gram negative isolates 141 (22%) were AmpC producers and found to be 100% resistant to co-amoxiclav, cefoxitin, ceftazidime, cefotaxime, cefuroxime, cefixime, ceftriaxone, cefpodoxime, gentamicin, amikacin and aztreonam. Less resistance was observed against cefepime (30.4%), sulbactam-cefoperazone (24.8%), piperacillin-tazobactam (10.6%), ciprofloxacin (20.5%) and meropenem (2.1%). All the isolates were found sensitive to imipenem. The patients harbored AmpC β-lactamases were on various interventions in which intravenous line was noted among (51.1%), naso-gastric tube (37.6%), ambu bag (8.5%), endotracheal tube (3.5%), ventilator (2.1%) and surgery (0.7%).

**Conclusion:**

Extensive use of invasive procedures and third generation cephalosporins should be restricted to avoid the emergence of AmpC beta-lactamases in neonates.

## INTRODUCTION

Beta-lactamases are the bacterial enzymes produced by a number of bacteria that provide resistance to beta-lactam antibiotics which include penicillins, carbapenems, cephalosporins and monobactams. AmpC beta-lactamases hydrolyze all the broad spectrum cephalosporins such as cefoxitin, cefotaxime, ceftazidime and ceftriaxone.^1^ These enzymes are typically present in Gram-negative bacteria which include *Escherichia coli*, *Klebsiella* species, *Salmonella* species, *Shigella*, *Enterobacter* species, *Citrobacter* species, *Pseudomonas aeruginosa, Serratia marcescens*, *Providencia, Proteus mirabilis and Morganella morganii*.^2^

There is a lack of standard method for the detection of AmpC beta-lactamases however several different methods are being used for AmpC screening. The three-dimensional test is one of the methods for AmpC detection in which cefoxitin and ceftazidime or cefotaxime are used as indicator drugs.^3^ Another method known as Inhibitor Based Method, is used for the detection of AmpC beta-lactamases. In this method boronic acid is used as inhibitor of AmpC enzyme.^4^

The most frequent bacteria causing neonatal sepsis are *Klebsiella pneuomoniae*, *Acinetobacter baumannii*, *Escherichia coli*, *Enterobacter cloacae*, *Citrobacter diversus*, *Pseudomonas aeruginosa*, *Streptococcus pyogenes* and *Staphylococcus aureus*.^5^

Gram negative bacteria have developed high level of resistance to third-generation cephalosporins. The carbapenems, sulbactam and amikacin are used to treat neonatal sepsis caused by AmpC producing strains but if mutations occur in organisms, they can become resistant to carbapenems as well.^1^ Boronic acid can be used as AmpC beta-lactamase inhibitor.^4^ Multidrug resistance can be avoided by the restricted use of third-generation cephalosporins in neonatal sepsis.^6^

Major risk factors included indiscriminate use of third generation cephalosporins, prolonged stay in hospital and various invasive procedures. The aim of this study was to determine the frequency of bacteria in neonatal sepsis and to detect the presence of AmpC β-lactamases among them along with their antibiotic resistance pattern.

## METHODS

This present study was conducted at Microbiology Department of The Children’s Hospital and The Institute of Child Health Lahore, Pakistan, during September 2011 to June 2012. A total number of 1,914 blood samples were processed and the sample collection was consecutive and only one clinical isolate per patient (non-repetitive) was included in the study.

The blood samples collected in the brain heart infusion broth were incubated at 37°C. After a period of incubation the cultures were further processed on Blood and MacConkey agar plates. The bacterial identification was done on the basis of colony morphology, fermentation of lactose, oxidase test, Gram’s staining, biochemical tests, API 20E and API 20NE (bioMerieux, France).^7^

**Fig.1:**
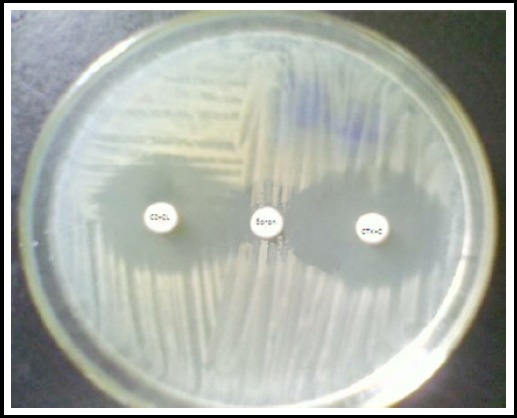
Detection of AmpC β-lactamase using inhibitor based method.

Isolates were screened for AmpC β-lactamase production by disc diffusion method as described by Clinical Laboratory Standards Institute (CLSI).^8^ The isolates resistant to ceftazidime, cefotaxime and cefoxitin were screened positive for AmpC beta-lactamases and further confirmed by inhibitor based method using boronic acid disc. In this method ceftazidime-clavulanate (CAZ+CL) and cefotaxime-clavulanate (CTX+CL) were used. CAZ+CL and CTX+CL discs were applied on the inoculated Mueller-Hinton agar plate. Discs containing boronic acid were applied in the center at a 5-10mm distance from ceftazidime-clavulanate and cefotaxime-clavulanate. The isolate was detected as AmpC producer if there was keyhole formation (synergism) between any of the cephalosporin+clavulanate and boronic acid disc (Fig.1).^9^

Antimicrobial sensitivity testing was performed on Muller Hinton agar (90mm) for each bacterial strain. A suspension of each bacteria was made according to the 0.5 McFarland turbidity standard and swabbed over the surface of Muller Hinton agar. *E. coli* ATCC 25922 and *Klebsiella pneumoniae* ATCC 700603 were used as control strains. The isolates were tested with different antibiotics which included amikacin (30 µg), aztreonam (30 µg), cefixime (5 µg), cefotaxime (30 µg), amoxicillin-clavulanate (20/10 µg), cefpodoxime (30 µg), cefepime (30 µg), cefoxitin (30 µg), imipenem (10 µg), meropenem (10 µg), ceftazidime (30 µg), ceftriaxone (30 µg), gentamicin (10 µg), sulbactam-cefoperazone (75/30 µg), piperacillin-tazobactam (100/10 µg), ciprofloxacin (5 µg) and cefuroxime (30 µg). After applying these antibiotics on Muller Hinton agar, plates were incubated overnight at 37°C. After overnight incubation the diameter of each zone of inhibition was measured according to the CLSI guidelines.^8^ The clinical record of each patient was reviewed. The clinical data of the patients was noted for the various interventions which included intravenous line, naso-gastric tube, ambu bag, endotracheal tube, ventilator and surgery.

## RESULTS

Out of total number of 1,914 blood samples 636 samples showed growth of various bacteria. A total number of 54 (8.49%) Gram positive and 582 (91.5%) Gram negative bacteria were isolated. Among the Gram negative bacteria AmpC beta-lactamases were detected in 141 (22.0%) isolates. The most frequent AmpC β-lactamase producing bacteria were *Enterobacter cloacae* 80 (56.7%) followed by *Enterobacter sakazakii* 20 (14.2%) and *Escherichia coli* 14 (9.9%). The rest of the AmpC β-lactamase producers were *Klebsiella pneumoniae* 8 (5.7%), *Citrobacter freundii* 8 (5.7%), *Klebsiella oxytoca* 4 (2.8%), *Serratia marcescens* 3 (2.1%), *Acinetobacter baumannii* 2 (1.4%), *Pseudomonas aeruginosa* 1 (0.7%) and *Aeromonas hydrophila* 1 (0.7%) (Table-I).

**Table-I:** Distribution of AmpC β-lactamase producing bacteria isolated in neonatal sepsis (n=141).

Bacteria	Frequency (n)	Percentage (%)
Enterobacter cloacae	80	56.7
Enterobacter sakazakii	20	14.2
Escherichia coli	14	9.9
Citrobacter freundii	8	5.7
Klebsiella pneumoniae	8	5.7
Klebsiella oxytoca	4	2.8
Serratia marcescens	3	2.1
Acinetobacter baumannii	2	1.4
Pseudomonas aeruginosa	1	0.7
Aeromonas hydrophila	1	0.7

All of the 141 AmpC producing bacteria were resistant to co-amoxiclav, ceftazidime, cefotaxime, cefuroxime, cefixime, ceftriaxone and cefpodoxime. Resistance rate of AmpC producing bacteria to various other antibiotics have been shown in (Table-II).

**Table-II:** Antibiotic resistance of AmpC beta-lactamase producing bacteria

Antibiotics	Resistant (n)	Percentage (%)
Co-amoxiclav	141	100
Ceftazidime	141	100
Ceftriaxone	141	100
Cefotaxime	141	100
Cefuroxime	141	100
Cefixime	141	100
Cefpodoxime	141	100
Cefoxitin	140	99.3
Gentamicin	136	96.4
Amikacin	132	93.6
Aztreonam	92	65.2
Cefepime	43	30.4
Sulbactam+cefoperazone	35	24.8
Ciprofloxacin	29	20.5
Piperacillin+tazobactam	15	10.6
Meropenem	3	2.1
Imipenem	0	0

The various interventions in hospitalized neonates included intravenous line (IV) 72 (51.1%), naso-gastric (NG) tube 53 (37.6%), ambu bag 12 (8.5%), endotracheal tube (ETT) 5 (3.5%), ventilator 3 (2.1%) and Surgery 1 (0.7%) (Table-III).

**Table-III:** Frequency of various interventions among the AmpC β lactamase harbouring neonates.

Interventions	Frequency	Percentage (%)
Intravenous line	72	51.1
Naso gastric tube	53	37.6
Ambu bag	12	8.5
Endotracheal tube	5	3.5
ventilator	3	2.1
Surgery	1	0.7

## DISCUSSION

Neonatal sepsis is one of the main cause of morbidity and mortality among the neonates in Pakistan. Among the positive cultures 582 (91.0%) were Gram negative and 54 (9.0%) were Gram positive isolates. These results are in accordance with other studies where Gram negative bacteria caused sepsis in newborns were high in number 92.8% than Gram positive bacteria 7.2%.^10^ A study reported 80.4% Gram negative bacteria and 20.6% Gram positive bacteria in neonatal sepsis.^8^ Muhammad *et al*. worked on neonatal sepsis and reported different results but with the high frequency of Gram negative bacteria (54.6%) as compared to Gram positive bacteria (45.4%).^11^

Detection of AmpC beta-lactamases poses a challenge to microbiologists. In the present study AmpC beta-lactamase producing bacteria were 22.0%. In other studies the prevalence of AmpC producing isolates was 26.8%^12^ and 19.61%.^13^ Manchanda and Singh worked on the occurrence of AmpC beta-lactamases from clinical isolates of Gram negative bacteria and reported 20.7% AmpC producing bacteria.^14^ Another study conducted on the detection of AmpC beta-lactamase producing isolates reported 35.5% AmpC producers.^15^

In the present study *Enterobacter cloacae* and *Enterobacter sakazakii* were most prevalent AmpC producing isolates. It has been studied that resistance emerged more often in *Enterobacter* species against cephalosporins than any other bacteria when treated with broad spectrum cephalosporins.^1^ *Enterobacter* species were the most common nosocomial pathogen among Gram negative bacteria and was the cause of pneumonia in 11% of pneumonia cases.^16^

All the AmpC producing isolates were found to be resistant to co-amoxiclav, ceftazidime, cefotaxime, cefuroxime, cefixime, ceftriaxone and cefpodoxime. A study conducted for the establishment of antimicrobial resistance pattern of AmpC producing Gram negative bacteria reported resistance to cefoxitin (99.3%), gentamicin (96.4%), amikacin (93.6%), aztreonam (65.2%), cefepime (30.4%) and sulbactam-cefoperazone (24.8%).^15^ A study on AmpC beta-lactamases reported that all the AmpC producers were resistant to cefoxitin and aztreonam.^11^,^14^ In the present study AmpC producers did not show high resistance to cefepime and sulbactam-cefoperazone which is in accordance with other studies in which AmpC beta-lactamases were observed to be less resistant to cefepime and all the AmpC producers were sensitive to sulbactam-cefoperazone.^15^,^17^

In the present study ciprofloxacin resistance was found to be (20.5%) and majority of the isolates were sensitive to this antibiotic which is similar to a study where AmpC producing isolates were also found sensitive (30%) to ciprofloxacin.^20^ In the present study rate of resistance against piperacillin-tazobactam was 10.6% which is similar to a study in which susceptibility pattern of AmpC producing isolates was determined and they were found to be sensitive to piperacillin-tazobactam (78%).^18^

In our study resistance against meropenem was observed to be 4.9% and none of the isolate showed resistance to imipenem. It shows that the majority of isolates were sensitive to carbapenems. Herman and Beatrice, (2005) carried out a study on the antibiotic susceptibility pattern of AmpC beta-lactamase producing *Enterobacteriaceae* and reported carbapenems as most effective antimicrobial drugs for the infection caused by AmpC producing *Enterobacteria*.^19^ Another study also reported the AmpC producing strains highly sensitive to carbapenems.^20^

The patients who harboured AmpC beta-lactamase producing bacteria were on various interventions which included intravenous line (IV), naso-gastric (NG) tube, ambu bag, endotracheal tube (ETT), ventilator support and surgery. Among these interventions, frequency of intravenous line was higher 72 (51.1%) than others. In another study intravenous lines were observed among the risk factors associated with nosocomial bacteremia.^21^ Ambu bags were observed as the source of transmission of pathogenic organism from one patient to another.^22^ The use of ETT is also a cause of microbial colonization due to the formation of biofilms. ETT was considered as the significant cause of development of ventilator associated pneumonia.^23^ A study conducted to detect the correlation of infections with various invasive procedures in different units of hospital. The rate of infection due to ventilator was 0.44 per 1000 ventilator-days and central-line was 4.6 per 1000 catheter-days in the neonatal intensive care unit.^24^ Surgical procedures are also common cause of acquiring the pathogens inside the body. Invasion of pathogens may occur due to use of contaminated apparatus during surgery or handling the surgical wounds with contaminated hands and dressings after surgery. The invasive procedures are associated with sepsis and are the major cause of blood stream infections.^25^

Neonatal sepsis caused by AmpC beta-lactamase producing bacteria causes treatment failure and high rate of mortality. Meropenem and imipenem can be used for their treatment. Different epidemiological studies should be taken on in hospital settings to monitor the sources of infection. To avoid the complications, minimize the use of unnecessary invasive procedures. Moreover, indiscriminate use of broad spectrum cephalosporins should be restricted in hospital environment and antibiotic policy should be revised time to time to reduce the emergence of AmpC producing bacteria.
